# A design process for a 3D printed patient-specific applicator for HDR brachytherapy of the orbit

**DOI:** 10.1186/s41205-020-00068-3

**Published:** 2020-06-29

**Authors:** Ergys Subashi, Corbin Jacobs, Rodney Hood, David G. Kirsch, Oana Craciunescu

**Affiliations:** 1 Department of Medical Physics, Memorial Sloan Kettering Cancer Center, New York, NY USA; 2 Department of Radiation Oncology, Duke University Medical Center, Durham, NC USA; 3 Department of Pharmacology and Cancer Biology, Duke University Medical Center, Durham, NC USA; 4 Medical Physics Graduate Program, Duke University Medical Center, Durham, NC USA

**Keywords:** High dose-rate, Brachytherapy, Applicator, Patient-specific

## Abstract

**Background:**

This report describes a process for designing a 3D printed patient-specific applicator for HDR brachytherapy of the orbit.

**Case presentation:**

A 34-year-old man with recurrent melanoma of the orbit was referred for consideration of re-irradiation. An applicator for HDR brachytherapy was designed based on the computed tomography (CT) of patient anatomy. The body contour was used to generate an applicator with a flush fit against the patient’s skin while the planning target volume (PTV) was used to devise channels that allow for access and coverage of the tumor bed. An end-to-end dosimetric test was devised to determine feasibility for clinical use. The applicator was designed to conform to the volume and contours inside the orbital cavity. Support wings placed flush with the patient skin provided stability and reproducibility, while 16 source channels of varying length were needed for sufficient access to the target. A solid sheath, printed as an outer support-wall for each channel, prevented bending or accidental puncturing of the surface of the applicator.

**Conclusions:**

Quality assurance tests demonstrated feasibility for clinical use. Our experience with available 3D printing technology used to generate an applicator for the orbit may provide guidance for how materials of suitable biomechanical and radiation properties can be used in brachytherapy.

## Background

The delivery of high dose-rate (HDR) brachytherapy can be complicated by irregular tissue contours, lack of appropriate patient-specific applicators, and deformable changes in patient anatomy. Currently, the fabrication of patient-specific devices is expensive and labor intensive. Additive and subtractive manufacturing, commonly known as 3D printing, have evolved from a broad discipline focusing primarily in research and development to one that allows for rapid and affordable fabrication of high-precision devices [13–15]. Technical progress in polymer chemistry, computation, and printing hardware has enabled the use of individualized delivery devices in both brachytherapy and external-beam radiation therapy [1, 3, 10]. This report describes a process for designing a patient-specific applicator for HDR brachytherapy of residual recurrent choroidal melanoma of the orbit.

## Case presentation

### Clinical history

A 34-year-old man received proton-beam radiation therapy in 2003 for a 17.0 × 14.0 × 10.5 mm melanoma involving the left choroid and ciliary body. He was treated with proton-beam therapy to a dose of 70 Cobalt Gray Equivalent, in five fractions, over 10 days. In 2017, the patient was in a motor vehicle accident, which resulted in rupture of his left globe. He underwent enucleation of the left globe and was found to have recurrent melanoma. The patient healed well from surgery and a left eye prosthesis was fitted. However, over the next 9 months the patient reported that the prosthesis became progressively displaced and increasingly painful to wear. A diagnostic CT revealed a heterogeneous lobular soft tissue mass in the anterior and inferior left orbit measuring 27.0 × 26.0 × 19.0 mm. He underwent salvage left orbital exenteration in March 2018. Surgical pathology confirmed multiple recurrent melanoma with a positive inferior-medial surgical margin. Restaging imaging revealed no evidence of metastatic disease and he was referred for consideration of re-irradiation.

Written informed consent was obtained from the patient for publication of this case report and accompanying images.

### Applicator design

An applicator for HDR brachytherapy was designed in the AutoCAD Inventor Suite (Autodesk, San Rafael, CA) based on the latest diagnostic CT. DICOM structures were converted into stereolithography files using 3DSlicer [7]. The primary contours of interest were the patient’s surface and the PTV. The patient surface was used to generate an applicator with a flush fit against the left orbital cavity and a protruding horizontal surface 10.0 mm anteriorly from the supraorbital ridge. Support wings with a thickness of 5.0 mm were designed to extend superiorly and inferiorly by 15.0 mm and laterally by 60.0 mm. The wings were designed to be flush against the patient’s skin in order to provide a stable and reproducible fit.

**Fig. 1:**
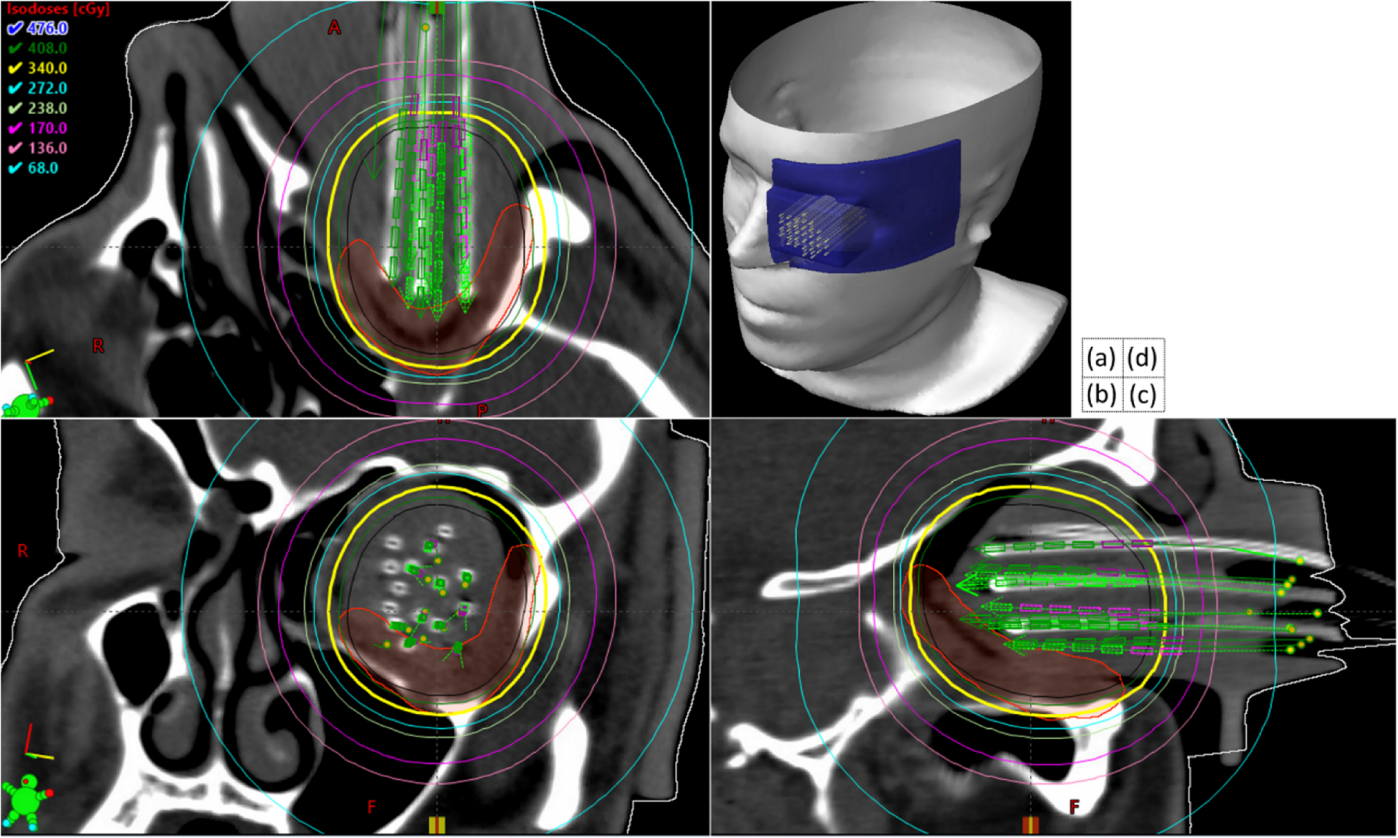
CT scan of the applicator immobilized in the orbit in (**a**) axial, (**b**) coronal, (**c**) sagittal, and (**d**) model view. Patient surface, PTV, and applicator are represented in white, red, and blue, respectively. Note that this CT scan contains the custom applicator in the patient’s orbit. Isodose lines are shown for a prescription of 340 cGy (yellow line)

**Fig. 2:**
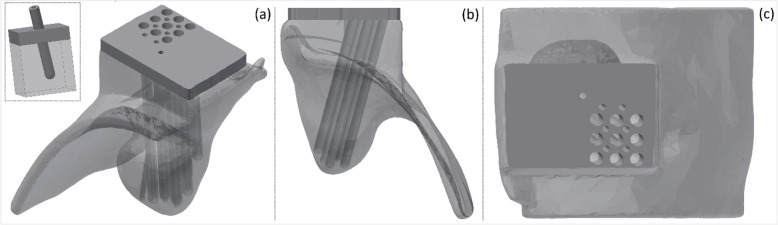
**a** Model view, **b** inferior-superior view, and **c** anterior-posterior view of patient-specific applicator for HDR brachytherapy of recurrent melanoma of the ocular orbit. The material for printing was selected based on similarity with biomechanical properties of human skin. Insert in panel (**a**) shows model for the design of the sheath of the source guide tube intended to prevent bending or accidental puncturing of the surface of the applicator

The involved left orbital surfaces, including the residual mucosa and soft tissue abutting the mass found on pre-operative imaging and the sites with positive margins, were contoured as the clinical target volume (CTV; Volume ~ 4.0 cm^3^) and radially expanded by 2.0 mm to generate a PTV. The PTV was used to devise channels that allowed for access and sufficient coverage of the target with the Ir-192 HDR source. The channels were constructed to fit an endobronchial HDR source guide tube with an outer diameter of 2.0 mm (Varian Medical Systems, Palo Alto, CA). While considering the size of the orbital cavity and the distance of the PTV to the cavity surface, it was found that sufficient target access would be provided when all channels were tilted 15° toward the patient’s right and 10° superiorly. For the most superior channel, patient anatomy did not allow for the 10° tilt. Under these constraints, the applicator was designed with 16 channels of varying length, ranging from 46.0 mm to 63.0 mm. The channels were organized in two rectilinear groups to minimize applicator size. The distance between the centers of the channels in each group was 9.0 mm. The tip of the channels, corresponding to the location of the first possible dwell position, was chosen to be at 5.0 mm from the surface of the orbital cavity. Figure 1 shows the applicator geometry overlaid on patient anatomy. The material for printing the applicator, an acrylic photopolymer (Polymerized TangoPlus and Agilus30 Family, Stratasys Ltd., Eden Prairie, MN), was selected based on similarity with the biomechanical properties of human skin [6], specifically using tensile strength and shore hardness. Note that these materials are not approved per the International Standard ISO-10993-1 as a biocompatible material. The applicator was covered in a sterile wrap to prevent any contact with patient skin. Given the flexibility of this material, a solid sheath with a thickness of 2.0 mm was designed as an outer support-wall for each channel to prevent bending or accidental puncturing of the surface of the applicator. The assembly was created using a J750 PolyJet 3D printer (Stratasys Ltd., Eden Prairie, MN) which allows for simultaneous printing of materials with varying physical properties. The primary applicator was designed as 80/20% mixing of polymers in the Agilus30/TangoPlus family; the sheath was 80/20% mixing of TangoPlus/Agilus30. Print time was approximately 20 h, while cost was approximately 400$. Figure 2 presents the design of the applicator and a model of the channel sheath. Photographs of the final 3D printed applicator are provided in the appendix Figure A1. Prior to use in the patient, the applicator was imaged with a helical CT scanner (120kVp, 1.0 mm^3^ isotropic voxels). The mean Hounsfield unit (HU) values were measured in a large region-of-interest in the applicator and found to be (mean ± std.dev.) 85 ± 11 HU, comparable to tissue-equivalent media. While previous research has highlighted the limitations of HU values in modeling the radiation interactions of 3D printed materials [4], the work of Baltz et al. has shown Agilus to be a reasonable tissue equivalent material based on CT and percent depth dose measurements [1].

### Testing and validation

**Fig. 3:**
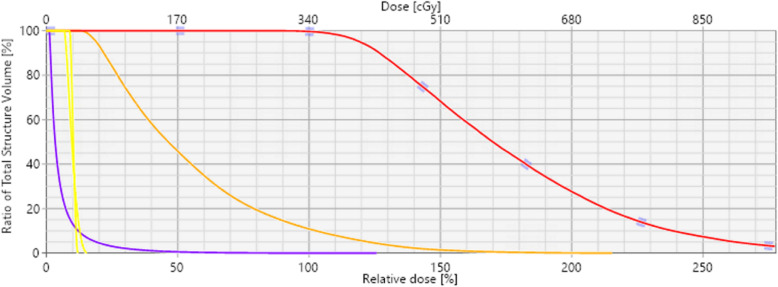
Dose-volume histogram for HDR plan with patient-specific applicator (shown for one fraction only). Red line represents PTV, orange is orbit bones, yellow lines are for right eye and lens, and purple line is brain

At the time of treatment simulation, the patient was immobilized supine with a custom thermoplastic mask and head holder. Serial axial CT images were obtained for treatment planning after placing the patient-specific applicator, covered in a plastic and sterile latex wrap, inside the left orbital cavity and securing it using self-adherent wrap. Figure A2 in the appendix shows the applicator inside the orbit at time of CT simulation. Treatment planning and dose calculation were performed in the Brachytherapy Planning module of Eclipse (Varian Medical Systems, Palo Alto, CA) based on the AAPM TG-43 [16] formalism, using Ir-192 at a nominal source strength of 10 Ci. The planned prescription was 3400 cGy, to be delivered in 10 fractions, twice daily, over five consecutive days [8]. Figure 3 shows the dose-volume histogram for the PTV, orbit bones, right eye, right lens, and brain.

An end-to-end dosimetric test was designed to determine feasibility for clinical use. Two calibrated pairs of optically stimulated luminesce dosimeters (OSLDs) were firmly placed on the surface of the applicator at two locations representing dose to PTV and another high-resolution CT was acquired. The clinical HDR plan was transferred to the CT containing the OSLDs in order to calculate the mean dose to the dosimeters. Figure A3 shows the registration between the CT of the applicator and the patient HDR plan. The applicator was then immersed in a water-filled container to mimic scattering conditions, and the clinical HDR plan was delivered. In the two sampled positions on the surface of the applicator, the mean difference in measured and calculated dose was 12% and 18%. For this setup, the standard error of the mean is equal to 50% of the standard deviation. This dose difference is in the range of published uncertainties for in vivo dosimetry in HDR brachytherapy [2, 12]. Finally, all ten fractions of the clinical HDR plan were consecutively delivered, amounting to a dose of at least 300 Gy to the surface of the applicator, and the applicator was monitored for structural damage. No damage was found over the course of 2 weeks.

### Treatment

A second CT simulation scan was obtained without the brachytherapy applicator for the purpose of generating an alternative stereotactic body radiotherapy (SBRT) plan. The orbital air density was assigned to water (i.e., 0 HU) in the treatment planning system to simulate a fluid filled cavity. A 4-arc volumetric modulated arc therapy plan with 6MV photons, utilizing superiorly oriented non-coplanar beams to avoid entry or exit into the contralateral eye, was created in Eclipse version 13.6 (Varian Medical Systems, Palo Alto, CA). The prescription dose was 2500 cGy, in 5 daily fractions.

Although most of the previously irradiated soft tissue was resected, the patient was consented for osteoradionecrosis and non-healing wounds. He was treated with SBRT instead of brachytherapy because the 3D printed material was not approved for biocompatibility and because filling the orbital cavity with sterile saline provided a reproducible bolus with fewer air gaps. While the surface of the applicator was generally flush with the orbit, we observe a maximum airgap of approximately 8.0 mm, comparable to previously published values [1]. This is illustrated in Figure A4 in the appendix which shows two slices from the CT of the patient fitted with the applicator. To confirm consistency with treatment planning, daily cone-beam CT was performed after filling the orbit with sterile saline.

## Discussion

In this report we describe a process for designing a 3D printed patient-specific applicator for HDR brachytherapy of the orbit. The physical properties of the polymers provided by the manufacturer are comparable to the relevant biomechanical properties of human tissue [6]. This similarity allows for a comfortable, stable, and reproducible fit in challenging locations in the body. When deciding on the material to use for 3D printing of brachytherapy applicators, biocompatibility and sterilization should also be considered [9, 11, 17]. Manufacturers are increasingly supporting the need for 3D printing of biocompatible materials that pass the ISO 10993-1 International Standard, as well as the United States Pharmacopeia standards of biocompatibility [5]. The medical physics and radiation oncology community have ongoing working groups to develop consensus guidelines for manufacturing and quality assurance of 3D printed applicators.

## Supplementary information

**Additional file 1: Figure A1.** Photograph of the final 3D printed applicator in inferior-superior view. This figure also demonstrates the placement of the OSLDs for our end-to-end dosimetric test. **Figure A2.** Applicator placement at time of simulation. **Figure A3.** Registration between the CT of the applicator and the patient HDR plan. One of the locations of the OSLDs is depicted by the white x-mark. **Figure A4.** Illustration of applicator fit at two CT slices demonstrating in panel **(a)** a flush fit and **(b)** the maximum airgap. While the surface of the applicator was generally flush with the orbit, we observe a maximum airgap of approximately 8.0 mm, comparable to previously published values by Baltz et al.

## Data Availability

The datasets used and/or analyzed during the current study are available from the corresponding author on reasonable request.

## Abbreviations

CT: Computed tomography
CTV: Clinical target volume
HDR: High dose-rate
HU: Hounsfield unit
OSLD: Optically stimulated luminesce dosimeters
PTV: Planning target volume
SBRT: Stereotactic body radiation therapy
