# Supplementary material for: A design process for a 3D printed patient-specific applicator for HDR brachytherapy of the orbit
Source: 3D Print Med. 2020 Jun 29;6:15. doi: 10.1186/s41205-020-00068-3 (PMC7322888; doi:10.1186/s41205-020-00068-3)
Supplement: Supplementary file 1 — Additional file 1: Figure A1. Photograph of the final 3D printed applicator in inferior-superior view. This figure also demonstrates the placement of the OSLDs for our end-to-end dosimetric test. Figure A2. Applicator placement at time of simulation. Figure A3. Registration between the CT of the applicator and the patient HDR plan. One of the locations of the OSLDs is depicted by the white x-mark. Figure A4. Illustration of applicator fit at two CT slices demonstrating in panel (a) a flush fit and (b) the maximum airgap. While the surface of the applicator was generally flush with the orbit, we observe a maximum airgap of approximately 8.0 mm, comparable to previously published values by Baltz et al. [file 41205_2020_68_MOESM1_ESM.docx]

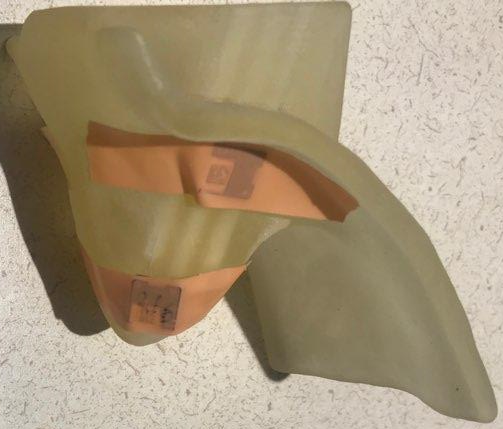


Figure A1. Photograph of the final 3D printed applicator in inferior-superior view. This figure also demonstrates the placement of the OSLDs for our end-to-end dosimetric test.


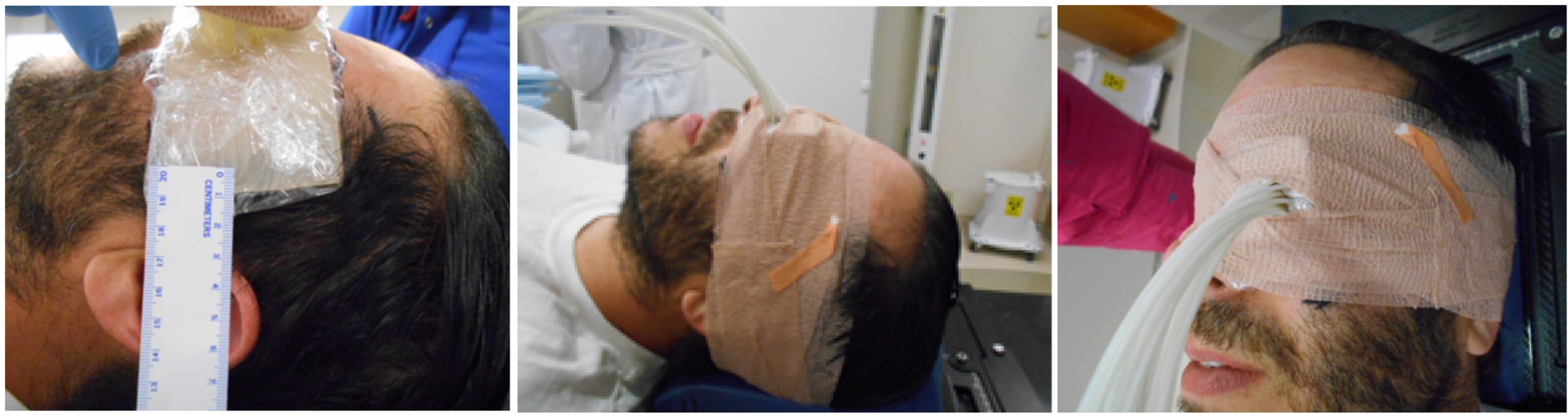


Figure A2. Applicator placement at time of simulation.


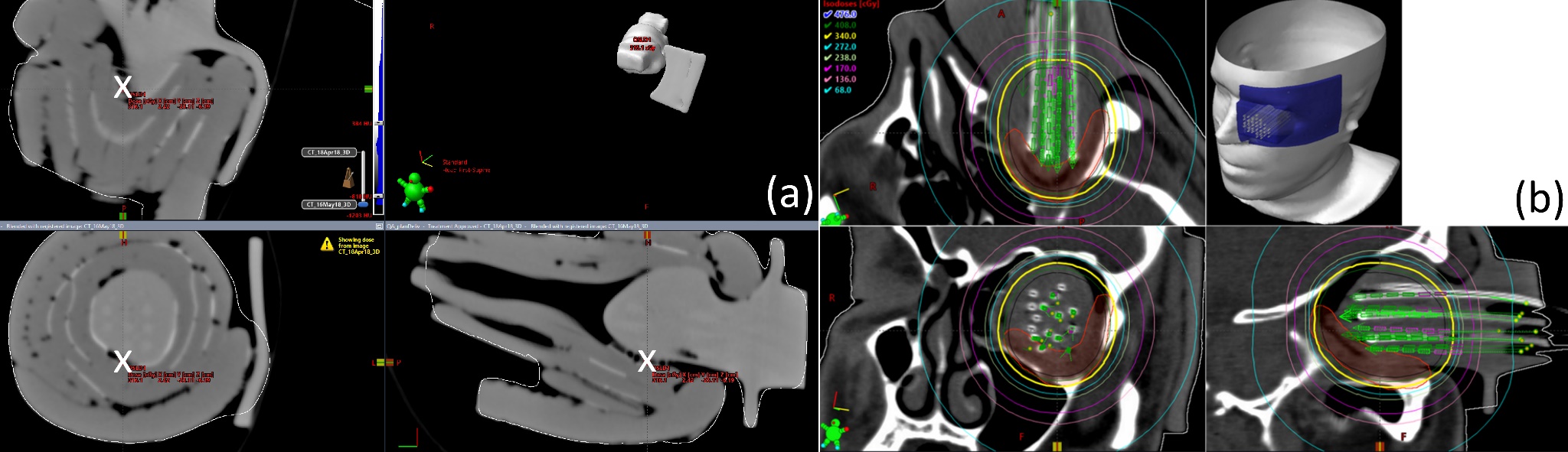


Figure A3. Registration between the CT of the applicator and the patient HDR plan. One of the locations of the OSLDs is depicted by the white x-mark.


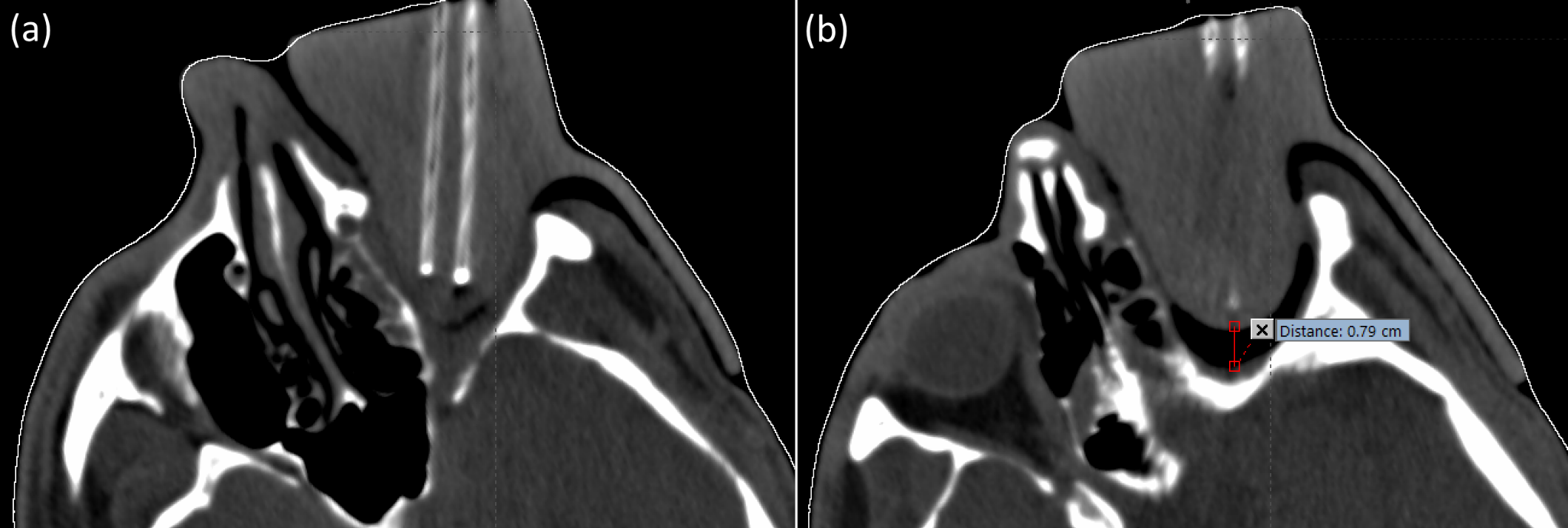


Figure A4. Illustration of applicator fit at two CT slices demonstrating in panel **(a)** a flush fit and **(b)** the maximum airgap. While the surface of the applicator was generally flush with the orbit, we observe a maximum airgap of approximately 8.0 mm, comparable to previously published values by Baltz et al.
